# Sales prices, social rigidity and the second home property market

**DOI:** 10.1007/s10901-023-10047-9

**Published:** 2023-06-14

**Authors:** Anne-Mette Hjalager, Michael Tophøj Sørensen, Rasmus Nedergård Steffansen, Jan Kloster Staunstrup

**Affiliations:** 1 University of Southern Denmark, Odense, Denmark; 2 Aalborg University, Aalborg, Denmark

**Keywords:** Real estate prices, Social status, Regional variation, Economic cycles, Governance

## Abstract

Second homes are much valued as recreational resources and also as important commodities on the property market. This study examines the trading patterns and regional price development of Danish second homes from 1992 to 2020. Second home sales volumes and prices reflect the general economic booms and busts and also the possibilities to rent out the property on sharing platforms. However, across regional clusters and over time, property price developments suggest a significant social rigidity in preferences and prospects. The investment and financialization logics and the underlying guiding conspicuous consumption behavior has not changed as an effect of the increased demand during the early phases of the COVID-19 pandemic. When controlling for factors such as house and land plot size, building year, location attractiveness the strong social class and spatial rigidity is reproduced in the data. The shifting of wealth accumulated in the second homes between generations supports the same tendency, and taxation does not rebalance regional effects. Accordingly, only to a limited extent does owning a second home contribute to social equality, even if some second-home owners and policy makers tend to think otherwise. Economic measures in planning and governance portfolios are found to be negligible.

## Introduction

Over the past decades, second homes have generated substantial academic interest, particularly regarding their significance as recreational and touristic resources (Hall & Müller, 2004, 2018; Müller, 2021; Roca, 2013). Second homes are objects of socio-spatial dynamic forces, whether in the micro universe of single groups of users, in the larger context of the community context in which the second home is embedded, or even more broadly in the ever-evolving leisure consumption cultures and markets (Keogh et al., 2022; Paris, 2009; Skak & Bloze, 2017). For individuals, driving factors for the acquisition of second homes are, for example, connected to obtaining access to natural environments, seaside freshness, and the possibilities for outdoor activities (Larsen, 2013; Overvåg & Berg, 2011). The composition or the comprehensive qualities, including the supportive infrastructures and neighborhood composition can add to the basic attributes and affect the overall attractiveness (Persson, 2014). However, money is also important. Second homes are investments not only in functional vacation capacities, but also in pecuniary matters, intermingled with social and financial value and assets. The study of the financial dimensions of second homes is dispersed over several intriguing aspects, some of which are also of importance for a prior understanding of how recreational real estate gains importance in wealth accumulation and, related hereto, as social status signifiers for the owners.

The purpose of this article is to examine second homes as property and investment objects. The assumption is that trading patterns and prices expose social values beyond the immediate qualities attached to the property and the surrounding environments. In the investment process, second-home owners may be interested in the prospects of short and long-term return of investment. But there are also underlying value systems that may be exposed in price developments when analyzed in a regional context. For this inquiry, the article aims to add substance to theories of “financialization” and to ideas in “conspicuous consumption”, in which second homes are regarded as a luxury commodity and simultaneously as objects of short or long-term speculative behavior.

The second home sector in Denmark serves as a case in this endeavor. The sector has national particularities and features that can support a generalization in the attempt to understand property as investment objects and class markers. There are more than 200,000 s homes in Denmark. They are owned by private persons, as commercial ownership of the most prevalent type of second homes is not allowed in Denmark. Around 29% of the population has access to a second home, either as owners or through being closely related to or friendship with the owners. To a considerable extent, second homes are used for family recreational activities across generations and kinships, also including friends (Knudsen & Knudsen, 2020; Larsen, 2013). For that reason, there is a strong emotional connection to second homes, and there is a tendency to regard this vacation accommodation resource as “democratic” in terms of accessibility, a symbolic expression of a society with a strong emphasis on egalitarian values, also when it comes to leisure opportunities. Policy makers and other opinion leaders express the wish to develop second homes as holiday resources, as they are so obviously preferred by Danish domestic vacationers from all social segments (Larsen & Laursen, 2014). Spatially detached from the first home, life in second homes is lived at a slower pace and with fewer of the urban pretentions and ambitions (Therkelsen et al., 2012). This second home narrative is ubiquitous, supported by the fact that the visual impression of most second home areas is fairly “low key”, with small and most often wooden houses and vegetation natural to the area requiring limited upkeep. As such, most second homes are clearly different from suburban homes.

Trading price analysis can assist in examining investment intentions and wealth accumulation. A main contribution of this article in the field of vacationing and tourism economy, which is, both nationally in Denmark and internationally, largely under-investigated. Evidence is needed to add to the discussion of some concealed social implications. Linking social aspects more clearly with economic ones can open for new planning discussions and policy scenarios and readdresses the controversies about egality and access to the leisure resource.

## Literature review

This article spans social and economic issues of second homes, with an emphasis on the latter, although the interrelationship between these matters is given major attention in the article. The scholarly literature can contribute with some crucial evidence on the specificity of second home value propositions. However, this literature review also addresses ideas and approaches in selected theoretical fields worth bringing into the inquiries about property trade. Thus, the literature review elaborates on property prices, long-term intergenerational wealth accumulation and financialization, and taxing. In these matters, there are links to touristic and recreational policies.

A much-treated issue in the international literature involves the spatial transformation of attractively located homes, where local ownership of residential property is transmitted to holiday houses and apartment space (Barnett, 2014; Gallent et al., 2003, 2017; Paris, 2009). Attached to this line of research is the focus on the displacement of original residents following from steeply increasing prices that do not match these inhabitants’ financial capacity. Consequently, an outmigration may take place (Müller & Hoogendoorn, 2013), although it may not, as migration depends on numerous factors (Marjavaara, 2009). An emerging spatial division of labor between the core tourism areas with high amenity values and not so attractive hinterlands, which serve as residential areas for those who work in the tourism industry, is part of the economic development in some, although not all, places. Research raises questions about whether the segregation trends can in fact be considered advantageous progressions. An argument discussed by, for example, Strapp (1988) is whether second home development can replace the decline in other types of tourism, and whether gentrification may enhance the local economy and stimulate job creation that can outweigh the decline in traditional trades, for example, in agriculture, because of rising property and land prices (Hoogendoorn & Visser, 2010). The research does not offer authoritative answers to these questions, and the discussion is whether the touristic transformation is the best option under the circumstances, when adverse alternatives may be a more comprehensive abandonment of the area (Overvåg & Berg, 2011).The rising property prices may be seen as a signifier of either success or failure, depending on the perspective (Collins, 2013; Hilber & Schöni, 2020; Hoogendoorn & Marjavaara, 2018).

Distance to urban agglomerations is found to be of critical importance for the level of obtainable sales prices (Back, Marjavaara & Müller, 2021; Hjalager et al., 2011). This suggests that convenience of use is a decisive matter for the selection of a second home. Modern lifestyles with shifting work obligations and social arrangements can require many short-term stays rather than longer holiday periods in the second home, which supports the claim for shorter travel distances. Despite this, the average distance between first and second homes has increased, indicating that prices of properties near urban agglomerations are becoming too high for the average household income. Improved infrastructure and relative gasoline price stagnation enable greater distance between the first and the second home (Hjalager & Staunstrup, 2009). During the pandemic, second homes became places where people could seek needed isolation (Müller, 2021; Zogal et al., 2022), and the property could also accommodate secure teleworking. This also refueled the discussion of the size, standard and comfort levels of second homes, which are often built mainly for summer use during shorter periods of time and are not supplied with sufficient insulation, heating, and other facilities. As noted by Hjalager, Staunstrup, Sørensen & Steffansen (2022), the pandemic spurred an intensification of building, rebuilding, and renovation of second homes, potentially also adding to a long-term trading value.

Many second homes are family owned and integrated into an inherited recreational capacity that is shared in a family and over generations. Also, younger generations tend be emotionally attached to second homes experienced during their childhood (Pitkänen et al., 2014). The second homes “store” intergenerational emotions and wealth (Pers et al., 2018). In parallel, second homes can be understood as commodities on the general property market, where sales volumes and prices are affected by and integrated with other categories of home ownership. The purchase of a second home depends on the individual’s availability of financial resources and possibilities to invest, but second homes also constitute elements of overall societally accepted and operational market-based wealth regimes. The property category is favorably included in public policies (Walters, 2019). Accordingly, property taxes, mortgaging rules, and direct or indirect state support can be, and are in many countries, construed in targeted ways to support or discourage average citizens’ wealth accumulation in real estate. However, some constellations of policies can unintentionally lead to speculative investments, as seen in the field of second homes (Hjalager et al., 2022). The second home market represents immanent societal inequality, as a second home will always, by definition, be additional to the first home (Soaita et al., 2020). The market mechanisms for second homes are affected by general affluence, interest rates, building costs, cost of transportation, and so on, and are thereby influenced by societal economic fluctuations (García, 2022). However, some owners see second homes primarily as objects of investment, in the past decade encouraged by rapidly rising property prices and low interest rates. In addition, second homes can largely be turned into a lucrative business through private letting (Keogh et al., 2022), which in Denmark is favored by tax deduction rules.

Price developments of second homes and the background for them are covered to some extent in Swedish research on the topic. Generally, prices for second homes have been on an upward trend, and Müller (2004) finds that the international access to the Swedish property market has enforced this development, but more so in the southern part of Sweden than the peripheral northern regions. More recent studies (Back, Marjavaara & Müller, 2021) can confirm this pattern, and the research points to an increasing spatial inequality, in which amenity-rich zones prosper more and obtain higher second home prices than hinterlands with less value in this regard. Second home property and prices are sensitive to economic fluctuations. García’s (2022) analysis demonstrates a sharp boom and also a rapid boost in the most popular areas, and he mentions credit opportunities as major reasons for the observed changes over time. The boost and busts in this respect are aggravated by speculative influence between non-professional second-home buyers, market anticipation, and mispricing. Nevertheless, amenity qualities and the social appreciation hereof are persistently decisive for the development of property values over time (Marjavaara & Müller, 2007).

Owners’ investment approaches and speculative property purchases not for own use are spurred by the sharing platforms on which property can be offered on the short-term rental market (Barron et al., 2021; Cocola-Gant & Gago, 2019). This is described as “financialization” of real estate (Kadi et al., 2020), where rent seeking within several parameters influences the property owners’ actions (Geisler, 2015). It goes hand in hand with a comprehensive political-economic and neo-liberal agenda, which celebrates radical resource utilization for economic gain (Doling & Richards, 2019; Hoogendoorn & Marjavaara, 2018). According to Morell’s (2020) studies on Mallorca, there is a subtle link between the gentrification of homes used for touristic purposes, the de-monopolization of hotels as primary providers of tourism accommodation, and the development of new semi-affluent classes with access to portfolios of property that can be used in a financially flexible manner. Without much other coordination than the ingrained belonging to a, for the time being, “winning strata”, the new investors tactically buy indebted owners’ properties, alter the physical appearance and social position of space, and apply an upward pressure on prices, both for rentals and for ownership. The financial linkages and mutual interdependencies between key investors, politicians, and rental companies are, according to Morell (2020), of critical importance for the creation or deepening of class differentiation in vacation space (Müller et al., 2021) and spatial inequality (Back et al., 2022). In the case of Denmark, Skak and Bloze (2017) find that younger generations tend to be more open to putting their second home on the rental market, suggesting an emerging unsentimental investment attitude regarding the property, thus also indicating changes in attitudes, which might manifest more clearly in the future.

As with primary homes, the second home is an object of distinct and continuous social valuing. Much of the sociological and anthropological research about the attitudes and perceptions of second-home owners and users can demonstrate that visits to the second home can imply a relaxation of the socially manifested appearances required in daily life: dress, food, activities, and so on. For a while, the holidaymakers can enjoy more primitive, simpler, and more spontaneous lifestyles together with friends and relatives (Persson, 2014), or engage with the community where the second home is located (Kaltenborn, 1998; Nordin & Marjavaara, 2012). The importance of socializing in combination with relaxation tends to be a main narrative (Larsen, 2013), but there is another, and perhaps partly contradictory, story to the social value of second homes. From a narrow economic and welfare perspective, a second home can be considered a luxury commodity, as it is unlikely to be a vital commodity in the same way as a primary home. As with other luxury goods and possessions (Belk, 1987), second homes can for some people be powerful signifiers of wealth, status, opportunities, and prestige, in other words objects of conspicuous consumption. Particularly well-located properties, for example, with a sea view or with large land plots, are “trophy” land, places where the elite can demonstrate taste, knowledge, and superiority (Geisler, 2015; Walters & Carr, 2019). The second home, its lavish appearance, architecture, and landscaped gardens display, and not least the location and the upmarket neighborhood, are social symbols. Hence, second home ownership can reinforce social and cultural disequilibria.

Second homes are elements in wealth accumulation both for professional investors and for private people and families who can set aside funds or save for later retirement, change of lifestyle, or other life choices. Paris (2009) examines the multi-residing, hyper-consuming classes, who have the means to shift between properties and can live a life in own homes but in different locations around the world. Comfort and the joy of experiencing different environments and climates are claimed to motivate the mobility of the multi-property owners, but the lifestyle also evokes the interest and admiration of peers and leads to the emergence of locational “hot spots” (Kadi et al., 2020; Müller, 2004). Critical comments about this type of second home mobility concern the owners’ lack of responsibility for the environment and for the regions where the multiple homes are located (Hiltunen et al., 2016; Xue et al., 2020). Owners travel more, use natural and limited resources with less care, ignore the need for a personal attachment to the overall maintenance of the destination, and they do not necessarily engage in co-citizenship matters (Barnett, 2014; Dykes & Walmsley, 2015).

Social and political movements argue for the availability of recreational resources for all, not only for the affluent classes. Not least, the Nordic second home literature (Müller, 2007; Persson, 2014) emphasizes describing the importance of egalitarian ideals and the accessibility of recreational landscapes as one of many manifestations of the welfare state. It also raises the discussion of whether simple second home lifestyle practices are in fact conspicuous non-consumption “luxury” in a new guise (Sørensen & Hjalager, 2020). Compared to other property categories, second homes receive less focus from policy makers (Back, 2022; Hall, 2015; Hjalager et al., 2022), and there is a lack of strategic choice in land use planning. Political countermeasures against gentrification and inflated prices could consist of an expansion of the overall capacity, for example, through prospective planning for new second home developments in areas not considered “posh” (Hilber & Schöni, 2020). In marketization thinking, the idea is to increase capacity, and then supply and demand will level out excessive profits, and accessibility will be ensured. Expanding seasons and rentals through tourism policy supported measures works along the same lines: incentives could be established through the adjustment of taxation rules (Larsson & Müller, 2019). The introduction and promotion of cooperative ownership opportunities and social housing models on touristic markets are also discussed (Slätmo et al., 2019).

This literature review demonstrates that the driving forces and implications of second homes have, over the years, acquired some research attention. However, there is a limited depth in the understanding of price developments in larger geographical areas and over longer periods of time. Likewise, there is a lack of insights into interlinking social mechanisms in the second home arena, where money and investment logics influence the spatial development. This study contributes to the closing of this gap.

## Methodology

The data source for the study is systematic property data for all second homes in Denmark, retrieved from the Danish Building Register (BBR), supplemented with information from the public sales and assessment register and the cadaster (SVUR). The unit of access is the second home as a clearly defined property. The approach in this study is quantitative, and trade volumes, prices, and locational data represent indicators for key issues and concepts in the study. The register does not include demographic or economic data on the owners.

Second homes are subject to special regulation in Danish planning law, and they have a specific category in the building register. Second homes cannot without permission be used for full-time residence, and regulation is quite rigid, which implies a high confidence in the reliability and usefulness of the data for this purpose. This research project has access to all 220,000 s homes in Denmark. All second homes are included that have, during the period between 1992 and 2020, been traded or shifted to family members via sales and inheritance. The database consists of 330,000 property trade events.

The study concentrates on second homes in areas that are dedicated to the purpose, that is, with a planning zone category of “second home zones”, as the regulation of second homes is different from other housing types (all-year residential villas and flats, and there is no risk of mixing housing purposes) (Barke, 2008). The second home zones constitute the dominant type of location in Denmark for second homes. Even if there is variance on many parameters including attractiveness and standards, the properties in the data set are what can be considered the “normal” type of second homes. Through the data validation processes, fewer than 1000 houses have been excluded, mainly houses with very disproportionally large land sites or house sizes, or second homes for which the other parameters were obviously incorrect, for example, age indicated for the building. Land sites dedicated to second homes but without buildings are not included in this study. The amount and the general quality of the data was considered adequate and accurate for a robust analysis.

The register includes variables about the year of trading of the individual second homes and the sales prices. There is information about the characteristics of trading, where the main forms are “normal” trading on the open market, shifted ownership through inheritance (registered for tax control purposes), and forced sales. There is a very small remaining category, around 1%, which includes, for example, property in compulsory arrangements. Variables chosen for this article also contain data about the specific locality (GPS coordinates and municipality) of the individual building.

A spatial categorization has been established through an iterative process. It is based on the 78 municipalities (out of 98) that have dedicated second home zones. A first test was undertaken on the 78 municipalities alone. Then, supplementing prior knowledge about the Danish tourism landscape, a regional aggregation in five groups was applied. The groups frame different categories of second homes in terms of distance to urban agglomerations, main natural features and importance on the rental market:

- The Copenhagen metropolitan area: Second home is in fair driving distance from the population agglomeration in Copenhagen and has a tradition for tourism, whereby the owners tend to commute from the second home during a long summer period. The areas are characterized by a relatively high density and a significant variety of house sizes and qualities.
- Zealand and Falster: Longer distances from Copenhagen, typically lower social tier second homes. The density is higher, houses typically smaller and lower in quality.
- Funen, the southern islands and Bornholm. An area where second homes are found more rarely and are a less recognized vacation resource.
- Jutland’s west coast: Regarded as a major holiday area for Danes and on the rental market. The west coast contains large natural areas and extensive outdoor opportunities. Second homes are on average bigger and located on larger land sites than in other parts of the country.
- Rest of Jutland. Areas of high variety, some located in the vicinity of larger cities, with weekend and holiday use combined. The east coast is characterized by pockets of significant natural value and outdoor recreational opportunities. The density of second homes is generally high, and there is variety in house standards and sizes.

The data from the study of trading events have been supplemented with other data regarding the location and the characteristics of the second homes, that is, size of building, size of land, year of building, year of latest rebuilding, height over normal sea level, and Euclidean distance to the nearest coastline. Simple correlations are provided to test the importance of these other factors.

The findings of the research will be presented below under the topical headlines: Trading activity, Regional patterns of trade, The development of sales prices, The geography of sales prices, and Other factors of importance for pricing.

## Trading activity

**Fig. 1:**
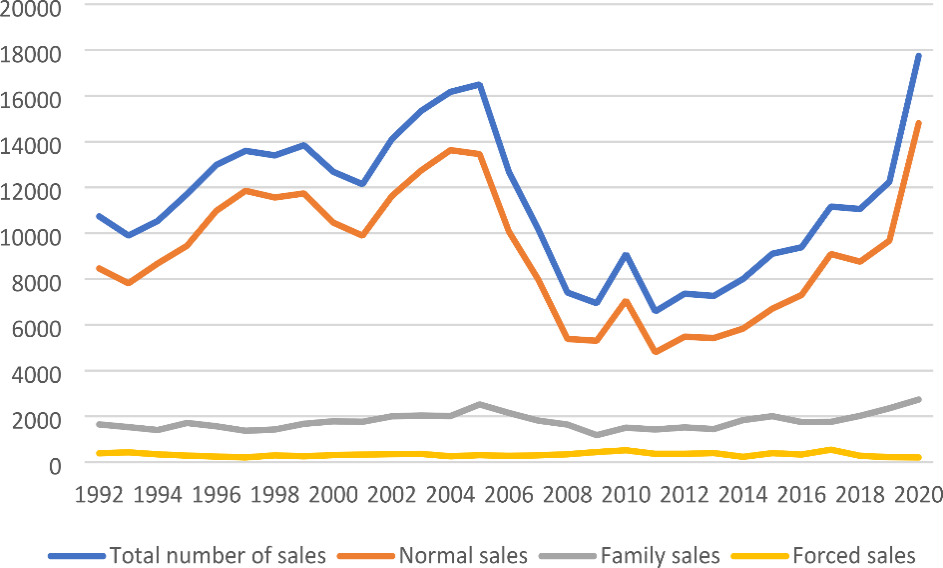
The number of second homes sold in the period 1992–2020, by type of trades

Over a period of 29 years, close to 330,000 trade events of second homes have taken place in Denmark. There is a distinction in the data concerning the nature of the transfer, and over the entire period, the large majority (80.6%) were trades advertised and promoted on the open market, as illustrated in Fig. 1. Trade between family members accounts for 15.6% of the trades, presumably mostly from parents to children, but other directions can also take place, however, data are not available on this. The amount of family trades does to some extent confirm that there is an intergenerational attachment to the property and that a second home may be considered a storage of capital over generations. The number of forced sales is low, around 1%.

Over the years, a significant variation can be observed, as illustrated in Fig. 1. An upward trend took place until the recession in 2008, when the trading activity took a remarkable plunge. From 2011, the number of trades increased again. During the first COVID-19 pandemic year, 2020, the sales frequency reached a maximum, but an intensification had already built up from the years before.

Normal sales trading on the commercial property market accounts for the highest level of activity, a pattern that is unchanged over the period. To some extent, the number of family trades reflects the same trend, and the recession also increased intergenerational trades. The economic constraints on (younger) family members can have led to a postponement of the takeover. In terms of taxation transfer to close family members (children and parents) is favored in the sense that the sales can take place according to the public valuation, which is significantly below the market price, minus 15%. This is of importance for taxation in connection with death duty at a later stage. As an effect of the digitalization of the taxation system, assessed values have not been increased since 2011. In 2024, at the scheduled restart of the system, assessed values are expected to increase quite considerably. The upward trend of family transfers may be an effect of speculative long-term attempts to avoid increased taxes.

Other trading forms account for very few sales. However, the number of forced sales increased after the recession. In 2006 and 2007, only 13 and 20 second homes, respectively, were forced sales, while in 2009 and 2010 the figures rose to 247 and 285, respectively. In 2020, the number was again low, at 27 trades. This is an indicator of the importance of economic cycles on the second home market.

According to the Danish law and exception from EU regulation, second homes cannot be purchased by foreign citizens unless they have a long-term and verifiable attachment to the country. This means that the second home market is by no means internationally oriented. Another measure implemented to ensure accessibility for Danish citizens is that there is an upper limit on the number of second homes that can be owned by a single person. Second homes can only be owned by private persons, not companies. These rules have been in operation for some time and can be regarded as countermeasures against extensive speculation.

### Regional pattern of trade

**Fig. 2:**
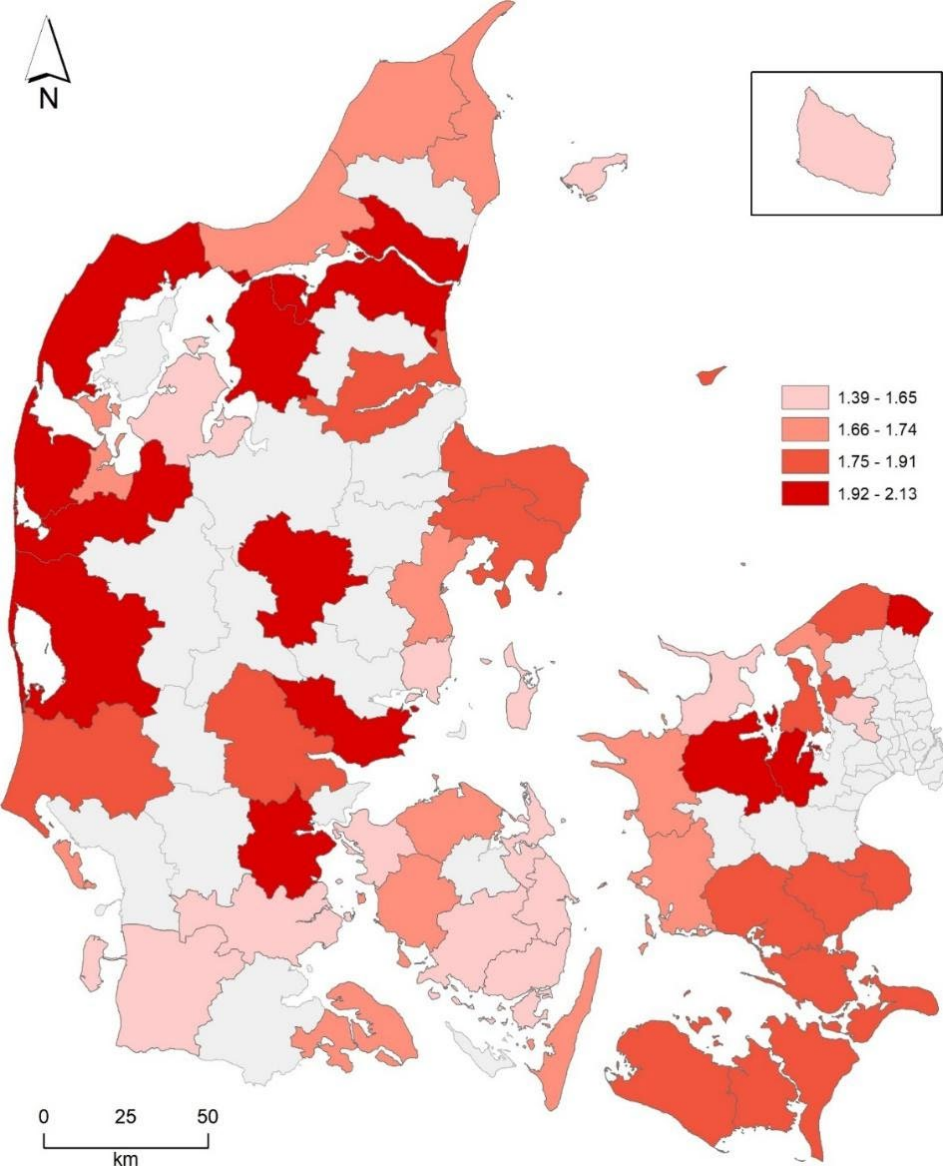
Sales turnover rates, number of sales per second home 1992–2020, by municipalities with more than 300 second homes

During the period 1992–2020 and for the whole of Denmark, every second home property was traded on average 1.78 times. It is of interest to know whether the market is slower or faster in some regions and areas than in others. Figure 2 shows municipalities and rates of turnover. Only municipalities with more than 300 s homes are included in the map. The lowest turnover rates appear in Bornholm (1.39 times) and Fåborg-Midtfyn (1.41 times). The highest are Lemvig (2.13 times) and Vesthimmerland (2.08 times). The variation cannot be considered extremely high, and it cannot immediately be explained by geographical location in the country.

Almost 30 years is a fairly long period, influenced by a fluctuating economy. In 2020, an extraordinarily high number of sales took place – nearly 20,000. The spatial distribution is not pronounced, as the shift of the second homes to new owners took place in all parts of the country with only some small variations. However, the number of shifts of property on the islands Bornholm and Samsø are lower, which might be an effect of the ferry transportation constraints during the pandemic. There is not any obvious predisposition for trading events in the areas close to the large cities, that is, near the Copenhagen metropolitan area or Aarhus. Accordingly, these data cannot in any clear manner support the belief about COVID-19 pandemic behavior, whereby second homes were purchased to acquire a safe work or leisure refugium close to the home address. As will be illustrated in greater detail below, trading behavior is not only affected by the location or such an extraordinary situation as the pandemic, but buyers are also influenced by the property price levels.

### The development of sales prices

There are data available on the sales prices of each of the almost 300,000 trades performed in the period 1992–2020. In the following analysis, only prices for normal trades and family trades are included. Forced sales and other trades account for very limited activity, and prices obtained vary considerably and beyond possible systematic explanation.

**Fig. 3:**
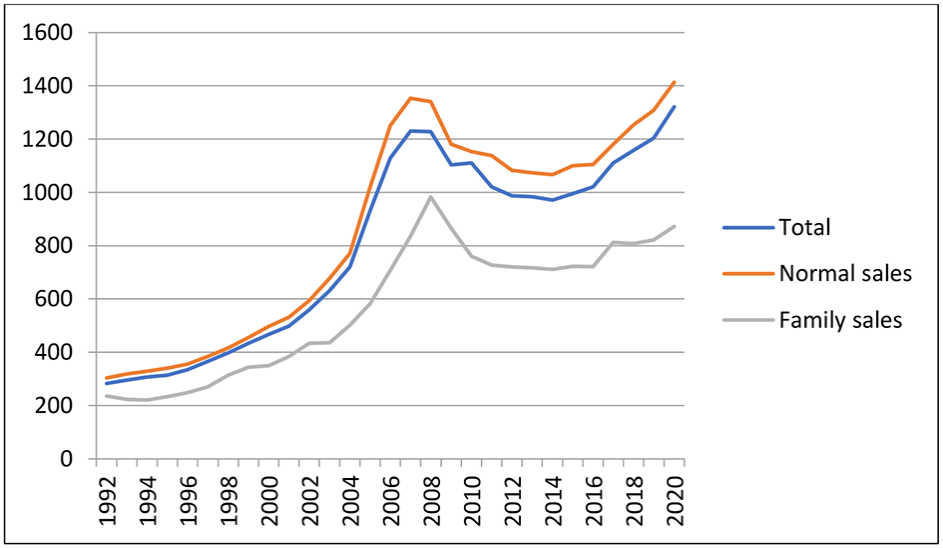
Average sales prices (DKK 1000, running prices) 1992–2020, by trading types

For the total period and for all trades, the average sales price (running price) amounts to DKK 772,000 (approximately 103,000 Euros). Figure 3 compared to Fig. 1 shows very convincingly how higher prices and lower numbers of sales were synchronized before 2008, and after 2008 the recession pushed the prices downwards. That accounts for both normal trades and family trades. After 2014, a new rising curve starts. The family trades do not follow the price development entirely, which is the effect of the outdated public property valuation system. This has created a disproportionate benefit for those families who have been able to use intergenerational transfer rules for second homes.

**Fig. 4:**
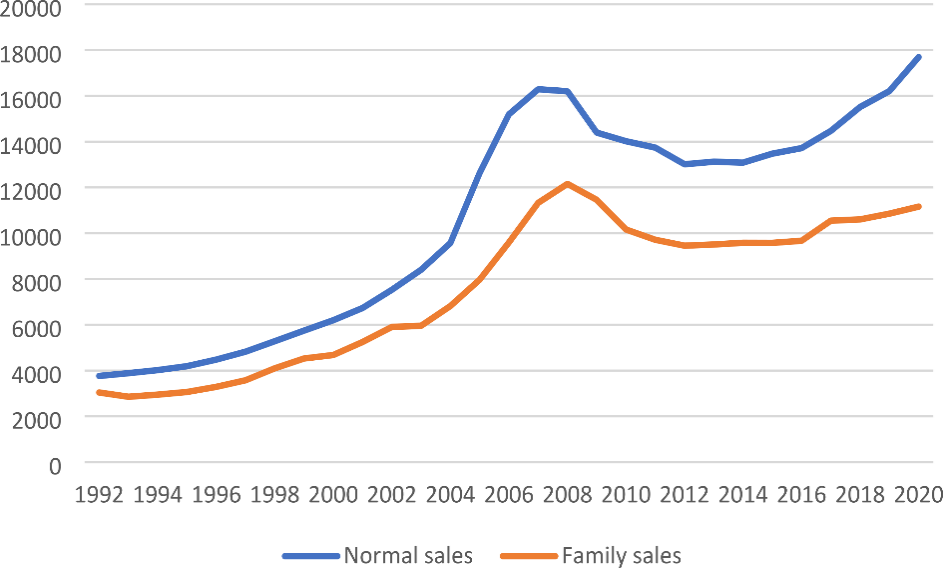
Average sales prices per square meter (DKK, running prices) 1992–2020, by trading types

The information above shows sales prices. For a new owner, it is critical in the choice and purchase situation to get an accurate knowledge of annual costs and the possibilities to obtain a mortgage. However, the price per square meter is another way to examine the price development, as it can be of importance for the price sensitivity. Figure 4 shows the average price per square meter on the normal market and in intergenerational trading. All prices have gone up over the years included in this analysis, and the pattern largely resembles that of total prices. But price sensitivity seems to be higher for the total price than for the square meter prices. Some buyers may have acquired smaller houses than planned to be able to afford a second home.

There are also lower square meter prices for the family transfers, and this type of sale also follow the general market, although not quite as markedly. Square meter prices have remained, for family acquirers, at an attractively low level, particularly in recent years.

### The geography of the sales prices

**Fig. 5:**
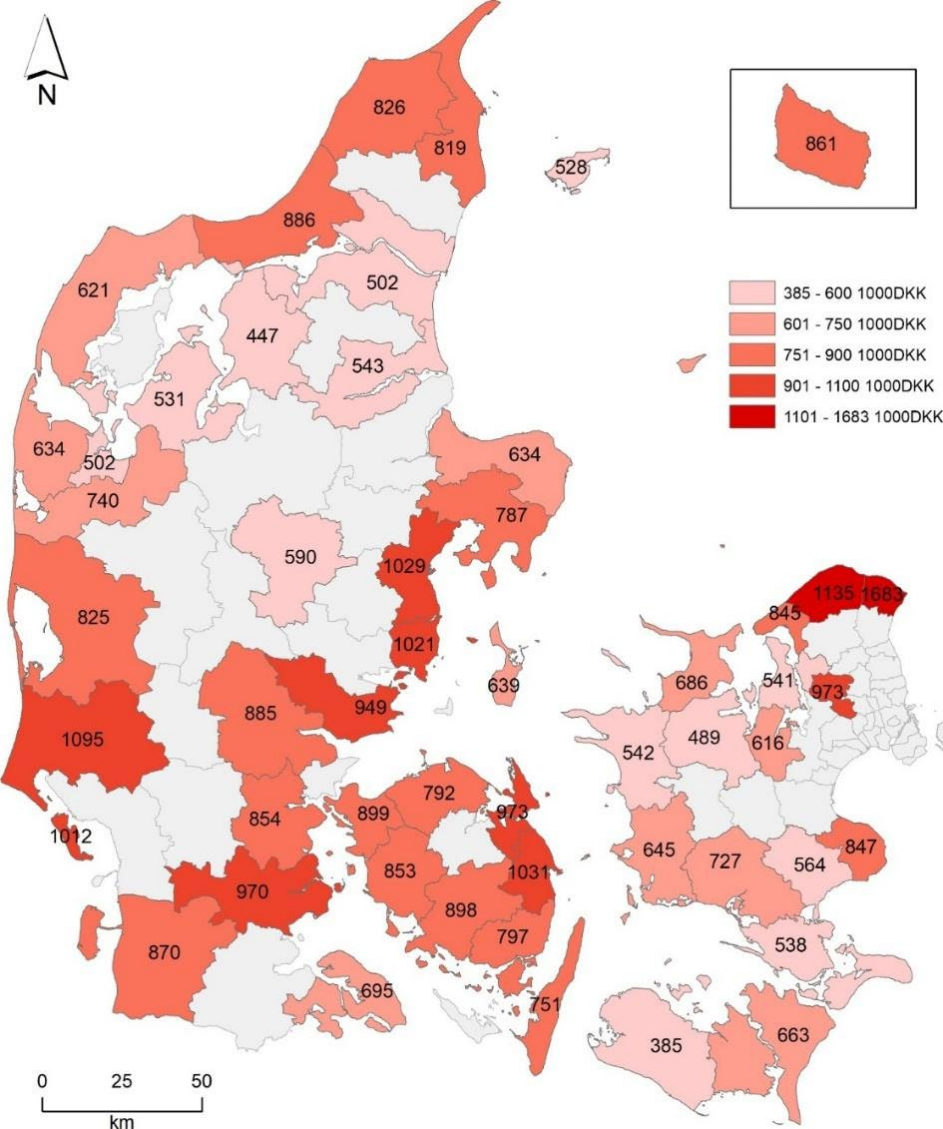
Average sales prices (DKK 1000) all second homes, by municipalities with more than 300 second homes

In particular, there are two spatial aspects of the sales prices that are interesting to pursue: average total prices in the regions and average square meter prices. The map in Fig. 5 illustrates average prices by municipality. As above, only municipalities with more than 300 second homes are included.

The municipalities of Gribskov and Helsingør are at the top of the price scale. These places contain very popular seaside resorts, such as Hornbæk and Gilleleje, and they are located within close distance to the population agglomeration in and around Copenhagen. Aarhus and the neighboring municipalities are also expensive areas, and, again, distance can be an explanatory factor, Aarhus being the second largest city agglomeration in Denmark. Due to nature and for recreational reasons, the West coast of Jutland lies high, except for Fanø and Varde. The West coast areas are main destinations for the commercial renting out of the second home capacity, particularly to visitors from the nearby German cities, such as Hamburg. At the other end of the scale are Lolland, Vesthimmerland, and Aalborg Municipalities, which have inland and eastward coasts, and a longer distance to the population centers.

Lower prices and higher turnover rates seem to go in parallel, and following from this, cheaper second homes are easier to sell than more expensive ones. However, the correlation is not significant.

In the following, prices for second homes are analyzed for five specific groups of municipalities. As introduced in the methodology, they represent different qualities of vacation landscapes. Data are treated for the latest sequence of years: 2015–2020, the period of upward trends after the recession. It can be observed in Table 1 that particularly second homes in or close to the metropolitan area have undergone rapidly rising prices.

**Table 1:** Average sales prices in DKK 1000 (running prices) for second homes, by municipality groups

	Average prices 1992–2020	Average prices in 2015	Average prices in 2020	Development 2015–2020
**Jutland’s west coast**	858	1101	1372	+ 24.6%
**The rest of Jutland**	680	890	1151	+ 29.3%
**Funen, the southern islands and Bornholm**	752	911	1174	+ 28.9%
**The Copenhagen metropolitan area**	981	1291	1899	+ 47.1%
**Zealand and Falster**	640	817	1123	+ 15.7%
**All**	772	995	1.321	+ 32.8%

Similarly, an analysis has been undertaken of the square meter prices, which can be seen in Table 2. An increase in the prices is seen following the same pattern as above for the total price. Second homes close to Copenhagen obtain better prices per square meter than properties in other parts of the country. However, the prices have gone up in Western and Southern Zealand, which have a slightly longer distance from the metropolitan area but are still reachable, for example, for brief weekend visits. Buyers on the heated metropolitan market may need to go for less expensive areas, and they are squeezed out from what are considered particularly attractive or exclusive destinations north of Copenhagen. Accordingly, buyers can purchase a second home in other parts of the country at a price that is acceptable for them.

**Table 2:** Square meter prices (DKK, running prices) for second homes, by municipality groups

	Average prices 1992–2020	Average price in 2015	Average price in 2020	Development 2015–2020
**Jutland’s west coast**	9637	12,619	15,645	+ 24.0%
**The rest of Jutland**	8656	11,204	14,887	+ 32.9%
**Funen, the south islands and Bornholm**	10,168	12,216	15,726	+ 28.7%
**The Copenhagen metropolitan area**	11,987	15,419	23,117	+ 49.9%
**Zealand and Falster**	8656	10,872	15,378	+ 41.4%
**All**	9799	12,351	16,586	+ 34.3%

As illustrated above, prices for second homes in Denmark increased during the long periods analyzed. But what about the boom year, 2020? In 2020, the prices were almost systematically double the average prices for the total period. A second home in Helsingør Municipality, a short commuting distance to central Copenhagen, between 1992 and 2020 gained on average an increase of DKK 1.68 million (224,000 Euros), and in 2020 the average price was DKK 3.3 million (440,000 Euros). In the much less prestigious Lolland Municipality, the sales prices between 1992 and 2020 were DKK 358,000 (48,000 Euros) on average, rising to DKK 614,000 DKK (82,000 Euros) in 2020.

Remarkably, the ranking of destinations does not change in any radical way over time, and not even an extraordinary market situation during the COVID-19 pandemic seems to have shifted consumers’ attitudes and preferences. Social association with vacation space and the related prices remains stable.

### Other factors of importance for prices

In the previous section, the location of the second home is seen as a determining factor for prices on the second home market. There are very clear differences between regions and municipalities, but evidently there are also other reasons behind the specific sales price of a single property, mainly the qualities of the individual second home and its immediate surroundings. Selected quality measures are correlated with average prices between 1992 and 2020, as seen in Table 3.

**Table 3:** Correlations between sales prices of individual second homes 1992–2020 and characteristics of the property

	Correlation with sales price	Correlation with square meter price
**The size of the house (square meters)**	0.265**	− 0.093**
**The size of the land site (square meters)**	0.069**	0.028**
**Building year of the house**	0.000	− 0.099**
**Year of latest rebuilding**	− 0.006**	− 0.026**
**Elevation above sea level, meters**	0.028**	0.023**
**Distance to the coast**	− 0.054**	− 0.060**

** Significant at 0.05 level

Most correlations are significant, although not strong. It is to be expected that large second homes are more expensive than small ones, and that is demonstrated in Table 3. The average size of second homes in Denmark is 80 square meters, and the variance is modest. In addition, the correlation is significant for the square meter price but in the opposite direction, and larger houses do not obtain higher square meter prices. This might signify decreasing utility, and those buyers overpay for a small house, for example, to get access to the vacationing capacity, and which they may later rebuild, expand, or resell on the market for something larger. Accepting a very small house may be a way into a prestigious area. Intermediaries on the rental market tend to be of the opinion that prices and rental occupation rates are higher for large houses, but this is not clearly reflected in relatively higher square meter prices.

The size of the land plot is also a positive factor for price. Land sites normally lie in the range of 1,000 and 3,000 square meters, and the variation is to some extent regionally determined, with the largest plots along Jutland’s west coast. The amenity values with open areas and low density are reflected in the prices, according to the correlation coefficients. This matches research on the lifestyles and holiday traditions of second-home owners, who seek tranquility in green environments (Larsen et al., 2014). This preference and the pricing mechanism are in contrast to the political or investor governed plans and ideas to ensure a higher density in second home areas (Hjalager et al., 2022). A densification can lead to an emotionally-based spatial resistance from owners and users (Hjalager, 2020). What is particularly crucial here is that this examination also demonstrates that the market value may be affected negatively if the density, in the buyers’ opinions, is increased too radically.

Perhaps counterintuitively, buyers pay more per square meter for old houses, possibly even houses in need of major renovations. A place-specific inquiry into this shows that the oldest houses built before 1950 obtain quite high prices. Locations in amenity-attractive landscapes or cityscapes, close to the sea, and houses that are likely to have architectural or heritage values are particularly highly priced. During the early era of second homes, some iconic seaside resorts were created, and they maintain high levels of attractiveness. This pattern stands in contrast to houses from the 1960 and 1970 s, when a rapid development took place, and those second homes are typically sold at lower square meter prices. The areas where they are located are also nowadays typically more uniform, and the houses and vegetation less outstanding, and they lie behind the first developed areas and thereby further away from the sea. Many of these second homes from the boom period may have reached an age where they need renovation. Renovation in such areas can add to sales prices, but not proportionally. Amenity values in the area must be supportive for the owner to experience an additional economic advantage.

As Danish second homes are typically located in the vicinity of the coast, there is a distinct risk connected to rising sea level, storms, and other climate-related events (Steffansen et al., 2023). Do risky locations affect sales prices negatively? Table 3 indicates a positive correlation between elevation above sea level and sales prices. Thus, second homes with a more elevated location are likely to obtain higher prices than second homes with lower elevations. There might be a risk assessment behind the pricing, but other analyses show that sellers and buyers very quickly tend to forget flooding events (Lautrup et al., 2021), and prices may not be permanently affected. In addition, some of the lowest priced second homes are located behind protective dikes in the municipalities of Lolland and Guldborgsund, and this can affect the overall perception of climate risks, even if the dikes cannot necessarily guarantee safety from any likely climate effects in the future. The association of elevation and price might therefore also indicate a preference towards sea views or seclusion from populated areas near the coast.

Hence, the last factor in Table 3 is the Euclidean distance in meters from the second home to the nearest seashore. The attractiveness of sea-based amenity values is reflected both in the total price and the square meter price. It is also supported by the typical marketing of second homes, in which agents mention distance as attraction factors, and a view to the sea allows a large price premium.

## Discussion

Evidence provided for this article demonstrates that in the long-term perspective (1992–2020), prices for second homes have quite consistently moved upwards. However, the second home market is clearly sensitive to economic recessions, shown as lower prices and a decrease in the number of traded second homes in the period 2008–2014. Also, special conditions for vacationers during the COVID-19 pandemic have affected the second home market with higher numbers of sales and radically higher prices.

Second homes can be and are sold in all parts of the country, but the price is an important means to ensure a demand. There are quite substantial price differences for second homes between different regions and types of space in Denmark. When it comes to rising prices, the winning region is the area in and close to metropolitan Copenhagen. Convenience of distance to the urban agglomeration is a factor, but second homes in this area are also connected to a considerable degree of conspicuous consumption, where the possession of property in highly esteemed ZIP codes is connected to positive social peer approval (Belk, 1988). For second homes, the region north of Copenhagen does indeed represent considerable amenity values, in terms of forests, lakes, and coastal landscapes, but such values can also be found in other regions. The composite effect of the reputation, (celebrity) media attention, the specific financial management of the second homes by the owners, and naturally and publicly governed landscapes contribute to the regional differences in price structures. Prices in the districts with similar distances to the metropolitan area, but in the less fashionable south, cannot keep pace.

The prices for family transfer have been artificially held down for a period as an effect of tax regulations. For future studies, it would be worthwhile to provide more detailed insight into family-based wealth accumulation in second homes than was possible with this study. It would be interesting to see whether financialization patterns are different from those properties acquired on the normal market, and in which locations inherited property has other class and social connotations.

What we have found remarkable is in this study is that over a long period of time, spatial differences in relative price levels are very stable. Regions and municipalities with the lowest prices in 1992 are the same as those in 2020. And likewise, the highest prices are found in the same regions and municipalities in 1992 and 2020. Accordingly, the social status of the housing rating is almost unaffected over a period of nearly 30 years. In this respect, second homes are very much in line with the relative price persistency of the residential housing market in Denmark (Larsen, 2014; Marx, 2020; Socaita et al., 2020).

The study can conclude that the characteristics of the specific second homes, size, land site size, year of building and rebuilding, climate risk exposure, and amenity value, understood as distance to the coast, are correlated with sales prices. Higher quality will normally give a better price, but for the guiding of price levels, location is more critical than these factors. This is also an indicator of social rigidity and conspicuous value determinants.

The findings in this study show that second homes have not been a motor for the economic advancement of the most disadvantaged regions, as many policy makers and opinion leaders have been hoping for and are claiming. Regions which are in the greatest need of growth stimuli are those where the accumulation and financialization are least pronounced, for example, the Zealand and Falster region. Expected spill-over effects on the local job market (retail, construction, commercial attractions, etc.) will not amount to the same in peripheral areas as in the affluent and high price areas. The inequality is supported further by the Danish mortgage system. Accumulated wealth in the assessed values in high price areas provides owners with a significantly better economic foundation to invest in second homes, that is, to rebuild, expand, and provide qualities that can create both rental value on the rental market, but also higher prices when the property is resold. The activity, as it takes place, results in a subtle reproduction of such social markers that contribute to the continuous high value of the property in some regions only, while others are constantly lagging behind, even when some land use improvements and advancements have been undertaken.

## Conclusion and planning perspectives

This article contributes to the understanding of how real estate (in this case, second homes) in Denmark contributes to the persistence of sustained long-term inequalities in wealth. The development over the past 30 years has not in any visible way been a motor for new strata of the population to acquire a position on the second home real estate market. In addition, it has become more difficult for outsiders and for people with modest start-up capital to enter the race for social status or wealth accumulation through financialized and speculative thinking and action (Jensen, 2021; Marx, 2020). While the first eras of the second home development between 1950 and 1970 allowed middle class and working-class people to purchase second homes, even with small incomes (Tress, 2002), this has not been an option to the same extent during the past 30 years. This claim is supported by Steffansen (2016), who points out similar trends in Norway and adds that expenses related to ownership have also increased considerably. Second homes are therefore no longer to be considered as objects that can contribute to accelerating a social equalization process.

As the study is based on property data, there is no evidence provided here about the particular economic reflections of the owners and buyers in connection with purchase, ongoing investment in the property, or in connection with sales events. However, second home purchasers seem to act mainly in an economically rational way, as discussed in the academic literature on this topic. They assess the qualities for own use of the property, including affectional issues, but also for resale and wealth accumulation opportunities, as far as the trading patterns reflect. The influence of amenities on the potential development of prices and value over a longer period is vital. Some municipalities allow higher densities in second home areas, possibility believing that this will enhance the touristic potential, but this policy is in contrast with the pursuit of social prestige and the long-term price premium for most property owners. A dilemma for policy makers is that more dense areas may lead to a relatively slower price development if amenity values are contested. This might limit the acceleration of social inequalities on the second home market, but measures in this direction might also trigger the needs for public investment and planning for compensating improvements to nature and infrastructural qualities in coastal zones.

Some of the second home areas established in the boom period between 1960 and 1980 and again in the 2000s were characterized by very loose and liberal planning requirements. Second homes in these areas have, over the past 29 years, had slower increases in price than areas from other periods. In other words, the lack of planning visions and care and painstaking governance in the lower strata of the second home areas have contributed to a consolidation of the “poshness scale” on the second home market. During the COVID-19 pandemic, owners increasingly expressed a need for more distinct and emphasized planning, and they demanded intensified collaboration and involvement to ensure not only an increase in functional and amenity qualities in the second home zones, but also measures to sustain value on the market. However, in Denmark there is still very little research-based debate on the pros and cons of the underlying financialization trends. Discussions about how to adjust planning measures in a way that affects pricing in a politically selected direction are still without sufficient depth.

In the popular (and populistic) agenda, second homes in Denmark are considered a kind of common resource, a “social capacity” for Danes, and there is continuous attention on whether and how this is available to the population, including those with modest incomes and investment possibilities. The contribution of this article is that it points to the complexity in the determination of market prices. If the peripheral areas with second homes with lower prestige on the social scale are to enhance their attractiveness, it will require a combined effort. Better planning, for example, with clearer and well-motivated building regulations, is one prerequisite. Planning should also include measures to adapt to climate changes. However, there is also a need to rethink mortgage, financing, and taxation systems. Such measures will hardly put an immediate end to speculative real-estate practices, if that is what policy makers want to see. But as Danish tourism is rooted in this built environment along the coasts, there is a need, as shown in the article, to reorient some of the policies.

## Data Availability

The study is based on register data in public administration systems, and they can be acquired by others on the same conditions.
